# Spatio-spectral optical fission in time-varying subwavelength layers

**DOI:** 10.1038/s41566-025-01640-1

**Published:** 2025-03-07

**Authors:** Wallace Jaffray, Sven Stengel, Fabio Biancalana, Colton Bradley Fruhling, Mustafa Ozlu, Michael Scalora, Alexandra Boltasseva, Vladimir M. Shalaev, Marcello Ferrera

**Affiliations:** 1 Institute of Photonics and Quantum Sciences, Heriot–Watt University, SUPA, Edinburgh, UK; 2 Elmore Family School of Electrical & Computer Engineering and Birck Nanotechnology Center, Purdue University, West Lafayette, IN USA; 3 FCDD-AMT-MGR, DEVCOM AvMC, Charles M. Bowden Research Center, Redstone Arsenal, AL USA

**Keywords:** Optical materials and structures, Materials for optics, Integrated optics

## Abstract

Transparent conducting oxides are highly doped semiconductors that exhibit favourable optical features compared with metals, including reduced material losses, tuneable electronic and optical properties, and enhanced damage thresholds. Recently, the photonic community has renewed its attention towards these materials, recognizing their remarkable nonlinear optical properties in the near-infrared spectrum. The exceptionally large and ultrafast change in the refractive index, which can be optically induced in these compounds, extends beyond the boundaries of conventional perturbative analysis and makes this class of materials the closest approximation to a time-varying system. Here we report the spatio-spectral fission of an ultrafast pulse trespassing a thin film of aluminium zinc oxide with a non-stationary refractive index. By applying phase conservation to this time-varying layer, our model can account for both space and time refraction and explain, in quantitative terms, the spatial separation of both spectrum and energy. Our findings represent an example of extreme nonlinear phenomena on subwavelength propagation distances, which provides new insights into transparent conducting oxides’ transient optical properties. This can be critical for the ongoing research on photonic time crystals, on-chip generation of non-classical states of light, integrated optical neural networks, ultrafast beam steering and frequency-division multiplexing.

## Abstract (web summary)

Nonlinear optical properties of transparent conducting oxides are explored through the full spatio-spectral fission of an ultrafast 93-fs pulse traversing a submicrometre time-varying aluminium zinc oxide layer in its near-zero-index region, providing insights into the use of these materials for integrated photonics, photonic time crystals and integrated neural networks.

## Main

The spatial engineering of the macroscopic refractive index is broadly exploited in integrated photonics for a myriad of applications including metamaterials, photonic crystals and optical gratings, whereas temporal engineering remains somewhat underexplored. Indeed, the modulation of optical signals via electro-optical and all-optical means is widely used in signal processing. However, the focus of these applications is only on the attained ON/OFF states rather than on the nature of light–matter interaction during the transition time.

The theory of wave propagation in non-stationary media developed starting from the concept of time-varying permittivity^1^. Experimental demonstrations of the effects of time-varying media on light were then introduced in plasma physics under the alias of photon acceleration. Within these settings, noticeable effects can be recorded as photons interact with a plasma undergoing an abrupt electron density transition^2,3^.

In general, when a propagating wave encounters a sudden change in the environmental refractive index, a phenomenon known as time refraction occurs^2–5^. This effect is analogous to what happens to a wave at a material interface in spatial dimensions, and it shifts the photon frequency to satisfy momentum conservation as opposed to the spatial case in which the wavevector changes to preserve energy (appendix A in the Supplementary Information). For time refraction, the condition *ω*_1_*n*_1_ = *ω*_2_*n*_2_ holds (where *ω*_1_ and *ω*_2_ are the angular frequencies before and after the time boundary and *n*_1_ and *n*_2_ are the indices before and after the time boundary, respectively).

This equation, in its differential form d*ω*/*ω* = d*n*/*n*, explicitly tells us that for a frequency conversion process to be relevant (that is, experimentally measurable), a large change in the refractive index is needed in a very short time span (appendix B in the Supplementary Information). For this reason, despite the enormous potentials and intrinsic scientific curiosity around time-varying materials, consistent experimental advancements have been lagging. In fact, typical real-world temporal index changes are either fast (for example, attosecond perturbations of bound electrons) or large (for example, material phase transitions), with these two attributes typically excluding each other^6^.

Recently, transparent conducting oxides (TCOs) have proven pivotal in the near-infrared region to overcome the previously stated trade-off between amplitude and speed. Within this spectral window, these materials possess a bandwidth exceeding 260 nm (refs. ^7,8^), and allow for a 100% change in the refractive index triggered by near-single-cycle pulses (6 fs)^8^. These remarkable results follow other previously reported key findings such as bandwidth-large frequency shifts^9–12^, unitary-change refractive index^13,14^, dual-colour hybrid nonlinearities^15^ and many others^16–19^, thereby creating the perfect playground for fully developing the potential of photon acceleration physics^20–22^. These materials also lend themselves towards the realization of photonic time crystals, which can also be theoretically exploited for light amplification^23–25^.

In this work, an ultrathin time-varying layer is attained by optically pumping a subwavelength film of aluminium zinc oxide (AZO) operating in its near-zero-index region. The optical excitation induces a large-refractive-index time gradient, which is first ascendent and subsequently descendent, thereby exerting opposite effects on the front and back of a synchronized probe. Therefore, the transmitted pulse is spatially split into two halves, each containing about half the overall spectral power. In addition, these two ‘fission products’ show strong spectral shifts, which are opposite in sign for both halves and centred at the near-zero-index carrier wavelength.

## Analytical model

Let us now lay down the basic mathematical steps to deduce an adaptation of Snell’s law for our specific case. Here a probe pulse trespasses a subwavelength thin film of a TCO layer whose refractive index varies in time under the effect of another ultrafast pumping pulse. Since the film thickness is about 1/30th of the spatial extent of the pump-pulse envelope, the maximum temporal index change is about 40 times bigger than the maximum spatial index change over the film thickness. For this reason, the medium can be considered uniform along the direction of propagation *z*.

In the geometric optics approximation, the electric field is written as *E* = *A*e^i*ϕ*^, where *A* is a slowly varying envelope and *ϕ* is a space–time-dependent phase. The frequency and wavenumber of the propagating beam are defined as $\omega \equiv -\frac{\partial \phi }{\partial t}$ and *k* ≡ ∇*ϕ*, respectively.

For this system, a ‘phase conservation law’ can be written at the interface as

1 $${\phi }_{{\rm{t}}}={\phi }_{{\rm{i}}}+{\phi }_{{\rm{l}}},$$

where *ϕ*_i_ and *ϕ*_t_ are the incident and transmitted phases of the probe, respectively, and *ϕ*_l_ is the additional phase contribution induced by the time-varying layer. This phase conservation law generalizes Snell’s law and allows the simultaneous breaking of both spatial symmetry (due to the physical presence of the TCO layer) and temporal symmetry (due to the time-dependent refractive index that the pump induces on the TCO layer). With this formalism, we can now specialize our formulas to both energy and momentum conservation, by taking the time and space derivatives of equation (1):

2 $${\omega }_{{\rm{t}}}={\omega }_{{\rm{i}}}-\frac{\partial {\phi }_{{\rm{l}}}}{\partial t}$$

3 $${k}_{{\rm{t}},x}={k}_{{\rm{i}},x}+\frac{\partial {\phi }_{{\rm{l}}}}{\partial x}.$$

Under the assumption that the spatial index change along the interface is negligible (in experimental terms, this corresponds to a pump pulse that is considerably larger than the probe), we simply have $\frac{\partial {\phi }_{{\rm{l}}}}{\partial x}=0$. It is worth noticing that this term is non-zero for the case of metasurfaces, which could lead to a further generalization of the modified Snell’s law reported previously^26^ (appendix A in the Supplementary Information). However, in the absence of a spatial phase gradient along *x* (that is, no metasurfaces), equation (3) gives

4 $${k}_{{\rm{t}}}\sin {\theta }_{{\rm{t}}}={k}_{{\rm{i}}}\sin {\theta }_{{\rm{i}}},$$

where *k*_i_ and *k*_t_ are the total wavenumbers of the incident and transmitted waves, respectively. Now, the crucial point is the expressions of *k*_i_ ≡ *ω*_i_*n*_i_/*c* and *k*_t_ ≡ *ω*_t_*n*_t_/*c*, which lead to

5 $$\frac{{n}_{{\rm{t}}}}{c}\left({\omega }_{{\rm{i}}}-\frac{\partial {\phi }_{{\rm{l}}}}{\partial t}\right)\sin {\theta }_{{\rm{t}}}=\frac{{n}_{{\rm{i}}}}{c}{\omega }_{{\rm{i}}}\sin {\theta }_{{\rm{i}}}.$$

At this point, equation (5) is well suited to represent the case of a plasmonic metasurface patterned on top of a nonlinear substrate^27^ (appendix B in the Supplementary Information). To complete the model adaptation to the case of an air–TCO optically pumped interface, we now need to add the assumption that *n*_i_ = *n*_t_ since the TCO layer is deeply subwavelength (that is, film thickness < effective wavelength), thereby obtaining

6 $$\left({\omega }_{{\rm{i}}}-\frac{\partial {\phi }_{{\rm{l}}}}{\partial t}\right)\sin {\theta }_{{\rm{t}}}={\omega }_{{\rm{i}}}\sin {\theta }_{{\rm{i}}},$$

from which one derives the angle of transmission as

7 $${\theta }_{{\rm{t}}}=\arcsin \left[\frac{\sin {\theta }_{{\rm{i}}}}{1-\frac{1}{{\omega }_{{\rm{i}}}}\frac{\partial {\phi }_{{\rm{l}}}}{\partial t}}\right].$$

**Fig. 1:**
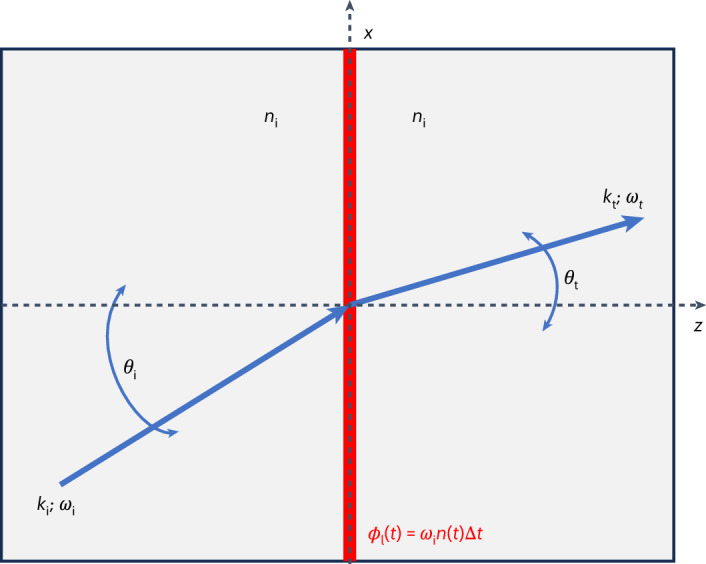
Spatio-temporal refraction. Adaptation of Snell’s law for a time-varying subwavelength thin layer, which can adequately model the case of optically pumped TCO thin films. In the picture, an optical ray trespasses a thin layer of non-stationary material immersed in a stationary and isotropic environment. The crossing induces a time-dependent phase shift, which is responsible for both spatial and spectral energy redistributions of the transmitted beam.

If there is no time variation in the refractive index (for example, switching off the pump), the contribution of the TCO layer to the phase would vanish $(\frac{\partial {\phi }_{{\rm{l}}}}{\partial t}=0)$, leaving *θ*_i_ = *θ*_t_, as expected. These two final equations can be associated to the scheme shown in Fig. 1, where an optical ray trespasses a subwavelength time-varying layer (representing our AZO thin film) immersed in an environment with a homogeneous and stationary index. It is worth mentioning that the fused silica substrate is completely ignored in this analysis, given its negligible contribution.

Now, an important step is specifying the phase *ϕ*_l_ accumulated by the incident wave during the interaction of the probe with the TCO layer. One natural guess is *ϕ*_l_(*t*) = *ω*_i_*n*(*t*)Δ*t*, where *n*(*t*) is the time variation of the refractive index in the medium due to the pump and Δ*t* is the interaction time of the probe with the layer. All this considered, the transmitted angle can be rewritten as a function of the index time gradient as

8 $${\theta }_{{\rm{t}}}\left(t\right)=\arcsin \left[\frac{\sin {\theta }_{{\rm{i}}}}{1-\frac{\partial n\left(t\right)}{\partial t}\Delta t}\right].$$

Let us now consider what happens to the wavelength of the incident radiation trespassing our time-varying layer. The transmitted wavelength can be written as a function of the index time gradient by taking the derivative of *ϕ*_l_(*t*) = *ω*_i_*n*(*t*)Δ*t* and substituting it into equation (2):

9 $${\omega }_{{\rm{t}}}(t)={\omega }_{{\rm{i}}}\left(1-\frac{\partial n\left(t\right)}{\partial t}\Delta t\right).$$

These last two equations will be recalled in the text several times to describe and explain all the reported results pertaining to the spectral and spatial redistribution of energy for an ultrafast pulse crossing a time-varying layer.

## Experimental settings

**Fig. 2:**
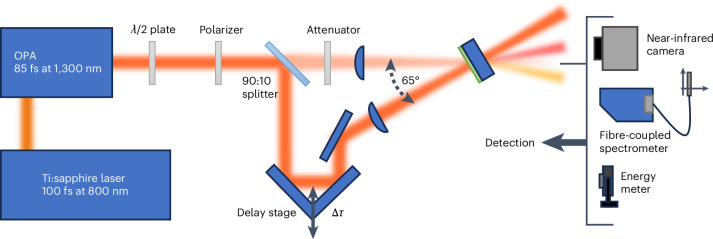
Experimental setup. Pump-probe apparatus operating in a degenerate configuration (that is, both pump and probe photons have the same photon energies). The pump pulse has the role of inducing a strong and ultrafast temporal change in the refractive index and turning our oxide film into a time-varying subwavelength layer. The schematic also shows the spatio-spectral fission of the probe pulse under opportune synchronization between the probe wave and the material response.

Experiments have been conducted via a pump/probe setup (Fig. 2) using 93 fs pulses (frequency-resolved optical gating characterization is discussed in appendix C in the Supplementary Information) at a repetition rate of 1 kHz and centred at 1,300 nm, which corresponds to the epsilon-near-zero crossover wavelength of our 900-nm-thick low-index (*n* ≈ 0.3) AZO film (the material characterization is discussed elsewhere^28^). The choice of the operational wavelength close to the crossover point is well justified by the relevant literature reporting on the enhanced nonlinearities in epsilon-near-zero media. The same applies to the decision of using a degenerate configuration for our experiments^29,30^. The pump beam is focused onto the sample at normal incidence with a peak power of about 1.8 TW cm^–2^ (well below the damage threshold^28,31^). The probe beam is attenuated well below the threshold for self-action nonlinearities and focused onto the sample at an angle of 65°. The condition of a steep angle of incidence for the probe is chosen for various reasons: (1) to maximize the local field enhancement^32,33^; (2) to size the material time response with respect to the probe duration for optimal spectral/spatial fission; (3) to maximize the relative induced change in transmission, thereby helping to detect spatio-spectral variations in the refracted signal; and (4) to provide the strongest angular shift according to equation (8) (appendix D in the Supplementary Information).

## Results

### Spatio-spectral fission

**Fig. 3:**
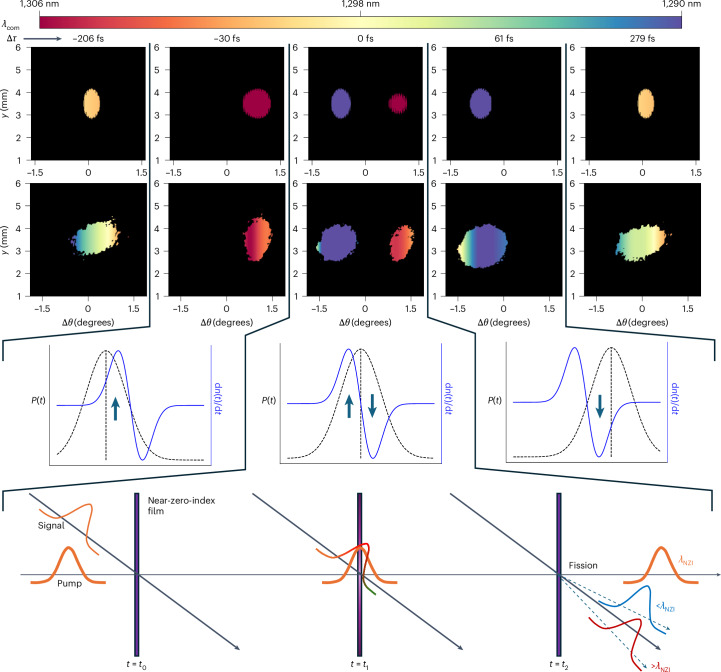
Full spatio-spectral information of the transmitted probe pulse as the pump-to-probe delay is tuned. The first two rows of panels show the probe-transmitted beam profiles (with the *x* axis recalibrated with respect to the deflection angle Δ*θ*) together with the associated spatial spectral distribution (heat map) for different values of the pump-to-probe time delay (Δ*τ*). The first row of panels pertains to a theoretical analysis performed using our model, whereas the second row shows the experimentally acquired data. Given the symmetry of the problem with respect to the incidence plane, only a horizontal scan of the spectral distribution is performed and one single wavelength value (that is, the centre of mass of the associated spectrum) is assigned for every given *x* (Δ*θ*), which is the same value for all the associated *y* values. The third row of panels shows black (dashed) and blue (solid) curves representing the probe temporal profile and the material response (d*n*/d*t*), respectively for specific pump-to-probe delays. Finally, at the bottom, a three-panel scheme represents, in a simplified manner, the full spatio-spectral fission of the probe pulse when Δ*τ* = 0, at which the probe and pump peaks are synchronized. For Δ*τ* = –30 fs, the leading edge of the probe experiences a positive refractive-index time gradient, which causes the probe to redshift by about 4 nm and deflect on the right by +1.15° with respect to the direction of the incident probe. For Δ*τ* = 0, the maximum pump-to-probe overlap is achieved and the probe splits into two halves with angular deflections of +0.85° and –0.7°, respectively. These two beams are characterized by different spectra corresponding to blueshifting and redshifting the two halves of the original spectrum with respect to its central wavelength. For Δ*τ* = +61 fs, the back of the beam experiences a negative-index gradient, and it is then detoured away from the original incident direction with a maximum angular deflection of –0.87° and the centre of mass blueshifted by about 7 nm. Finally, for Δ*τ* = 279 fs, the two pulses are no longer overlapped in time, and the transmitted probe is back to the unpumped case.

Following the previously outlined data analysis, we can plot the simultaneous spatial and temporal evolution of both energy and spectrum for a 93-fs quasi-transform-limited pulse trespassing an optically pumped subwavelength layer of AZO. This study is reported in Fig. 3, where four rows of panels show what happens to a pulse as it goes through a time-varying layer exhibiting two consecutive and abrupt changes in the refractive index, which are also opposite in sign. The outcome is indeed determined by the specific pump-to-probe delay Δ*τ*. The first row of panels shows the numerical predictions about the probe evolution trespassing the AZO layer for different pump-to-probe time delays. The second row of panels reports the corresponding experimentally acquired results. For the numerical evaluation, the probe beam is approximated using a spatially discretized *k*-vector distribution for a focused, spatially unchirped and transform-limited Gaussian pulse centred at 1,300 nm. This distribution is then used as input for equations (8) and (9), where the refractive-index temporal profile has been evaluated in the ‘Energy redistribution’ section.

From the first two rows of panels in Fig. 3, at large values of time delay (Δ*τ* = −206 fs, +279 fs), the probe does not interact with the refractive-index perturbation imparted by the pump, and thus, there is no shift (neither spectral nor spatial). Only a small wavelength deviation across the spatial width of the pulse is noticeable, which can be attributed to a small spatial chirp originating in the optical parametric amplifier. In this case, the pulse propagation direction is unaltered by the unpumped AZO layer. The quasi-transform-limited nature of both incident optical pulses is also proved by accurate frequency-resolved optical gating measurements (appendix C in the Supplementary Information).

If the pulse is slightly late (Δ*τ* = −30 fs) with respect to the material response (d*n*/d*t*), its front sees a positive gradient, as depicted in the schematic in Fig. 3 (first panel, third row). The transmitted pulse is then redshifted in wavelength (4 nm) and re-routed to the right (Δ*θ* = +1.15°) with respect to its original incidence direction (Fig. 3, second panel, first two rows). The dual case occurs if the incident pulse is mildly early (Δ*τ* = +61 fs) compared with the material response (Fig. 3, third panel, third row). In this case, it experiences a negative-index time gradient that deflects the pulse to the left (Δ*θ* = −0.87°) while blueshifting its spectrum (7 nm) (Fig. 3, fourth panel, first two rows). However, the most extreme situation pertains to the case for which the pump and probe are optimally overlapped in both time and space (Δ*τ* = 0). In this case, the probe is literally torn apart as its front and back are pulled in opposite directions by opposite index gradients. The two resulting halves are detoured away from the incidence direction (Δ*θ* = +0.85°; −0.7°) and being redshifted and blueshifted, respectively by almost the same amount as the previous separate cases (Fig. 3, middle panels (first, second and third rows) and the fourth row of panels).

It should be mentioned that this splitting is not perfect as there is a <10% residual optical power in between the redshifted and blueshifted beams. The origin of this ‘leakage’ stems from the simple observation that as the index of the time-varying film undergoes up and down transitions, the material assumes a time-invariant form for a brief instant when *dn*/*dt* = 0, thereby leaving some part of the probe beam unaltered according to equations (8) and (9). This residual power does not appear in our figure being below the set noise threshold. Given that the reported nonlinear spectral redistribution is linked to the temporal overlap between the probe time profile and the material response, appendix E in the Supplementary Information provides a related discussion.

### Energy redistribution

**Fig. 4:**
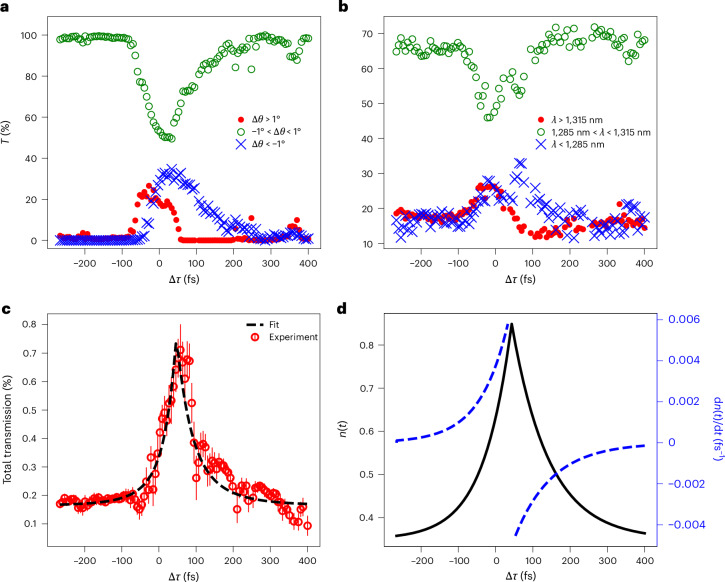
Power repartition and material response. **a**, Spatial power redistribution as a function of pump-to-probe delay Δ*τ* for three acquisition regions (that is, different areas on a near-infrared camera): percentage of transmitted power below –1° of deflection (blue crosses); between –1° and +1° (green circles); and above +1° (red dots). **b**, Power spectral density redistribution as a function of Δ*τ* for three acquisition spectral ranges: percentage of transmitted power below a wavelength of 1,285 nm (blue crosses); between 1,285 nm and 1,315 nm (green circles); and above 1,315 nm (red dots). **c**, Total percentage of transmitted power as a function of Δ*τ*. Data points with error bars represent the mean values ± standard deviation as calculated from 16 transmission measurements with identical experimental settings. **d**, Time profile of the effective index plotted as a function of Δ*τ* (black solid curve) and the corresponding temporal derivative (blue dashed line).

One limitation of the spatio-spectral analysis reported in Fig. 3 is that it shadows the acquired information about the energy redistribution mediated by the time-varying medium. To address this, we analysed the transmitted probe power repartition in both spectrum and space against the pump-to-probe delay. This repartition is shown in Fig. 4, where the acquired camera images and the spectra are partitioned into three distinctive regions, which allow to appreciate the redistribution of energy as the probe is affected by the time-varying layer. On one hand, the spatial power separation is considered in Fig. 4a where the three plots represent (1) the percentage of transmitted power below −1° of deflection (blue crosses), (2) between −1° and +1° (green circles) and (3) above +1° (red dots). On the other hand, the power spectral density separation can be described by plotting three curves (Fig. 4b): (1) the percentage of transmitted power below 1,285 nm (blue crosses), (2) between 1,285 nm and 1,315 nm (green circles) and (3) above 1,315 nm (red dots). By referring to Fig. 4a, we can, observe that the nonlinear spatial rearrangement of power for the transmitted probe pulse exhibits the same behaviour of its spectral redistribution as reported in the first two rows of panels in Fig. 3. By looking at both red dotted and blue crossed curves, we can clearly identify two regions, one in which the former is on top of the latter and the second in which the situation is reversed. In the first case, power is deflected from the centre towards the right side of our camera, as the pulse front experiences a positive-index time gradient. This is clearly represented by the rising of the red dotted curve and the contextual falling of the green circled curve. As we increase the time delay Δ*τ*, the red dotted and blue crossed curves intercept close to the point at which the green circled curve reaches a minimum, indicating an energy depletion of the central detection area and a spatial splitting pushing the energy on the periphery of the camera. As we continue increasing the time delay, we enter the second of the two previously identified regions (that is, blue crossed curve on top of the red dotted one), where the transmitted power is deflected towards the left side of our detection system under the effect of a negative-index time gradient. All the discussion in Fig. 4a can be applied in a similar fashion to the spectral redistribution reported in Fig. 4b, where the transient behaviour of the power spectrum is less clear due to the intrinsic difficulty in experimentally acquiring the spatial spectral information. A comment can be made on the limited signal-to-noise ratio in Fig. 4b. Here data are taken using a full spatio-spectral measurement, which is then reduced to lower dimensions (that is, integration over horizontal and vertical camera axes) to capture the essence of the process. More specifically, measurements were completed using an integrated spectrometer alongside automated stages. During data acquisition, we had to balance the noise floor of our measurement with the spectral sensitivity required to measure the blue- and redshifts—a task, which in our case, that was limited by the technical specifications of the used equipment. In practice, this issue can be overcome by employing a more advanced spectrometer (not available in our laboratory) featuring a lower noise floor. However, despite the limited signal-to-noise ratio, it is still evident from a direct comparison between Fig. 4a,b that the spectral power redistribution follows the same trend previously described for the angular power redistribution.

Finally, by acquiring information about the total percentage of transmitted power, the plot in Fig. 4c is attained, which shows an increase in transmission by over 300%. This apparent increase in the transmitted power is due to a reduction in the reflection at the interface under optical excitation, which leads to a temporary reduction in the air–AZO index contrast, as well as helping to highlight the energy splitting through the time-varying layer.

The nonlinear index modulation (Fig. 4d) was recovered from the transient transmittance shown in Fig. 4c. First, the relative transmission data are converted into absolute values of transmission using the previously reported linear transmission measurements for this same film^28^. An exponential rising index with a time constant of 75 fs (driven by the temporal profile of the Gaussian pump) and an exponentially falling index with a time constant of 100 fs (due to the relaxation of hot electron nonlinearities) are fitted to the transmission data (the transfer matrix approach is used to convert the fitted indices into transmission values). Both these time constants define a material time response that is slower than the pump temporal width, as expected. Additionally, the extremely low absolute transmission of the probe is due to the large angle used in the experiments and the remarkable index contrast between air and AZO. The imaginary index is held constant at the linear value in this analysis.

### Angular deflection

**Fig. 5:**
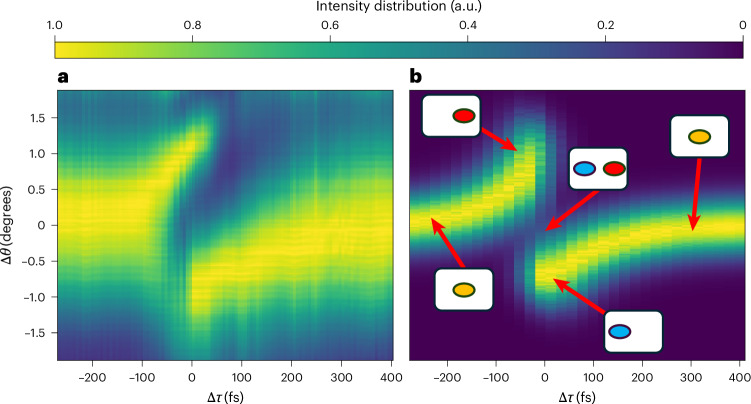
Power distribution as a function of deflection angle and pump-to-probe delay. **a**, Heat map of the angular intensity distribution as the pump-to-probe delay Δ*τ* is tuned. The picture displays the normalized heat map of intensity versus Δ*τ* and angular shift Δ*θ*, and it is attained by integrating the camera pixel intensity **I**_**c**_ (Δ*τ*, Δ*θ*, *y*) along the *y* axis. **b**, Theoretically calculated normalized angular intensity distribution. The second heat map is attained using the same numerical approach and parameters described in the ‘Spatio-spectral fission’ section, where integration has been performed on both spectrum and the *y* coordinate. The set of five little icons within the picture marks the temporal delays corresponding to the spatio-spectral plots reported in Fig. 4.

The normalized angular intensity distribution of the probe as a function of Δ*τ* is shown as a colour map in Fig. 5a. When reading this figure from left to right (corresponding to increasing values of Δ*τ*), we initially operate in the linear regime, with no overlap between the pump and probe and no observable angular deflection. As Δ*τ* progresses, the pump and probe start overlapping in time, and we enter into the redshift-dominant phase, which is accompanied by a positive angular deflection. Next, at a pump-to-probe offset close to zero, a clear spatial splitting regime appears, where in time, half of the probe (front of the pulse) overlaps with a positive-index gradient and the other half (back of the pulse), with a negative gradient. This regime stays dominant for about 45 fs (measured as the duration within which the absolute intensity difference between the right- and left-deflected beams is <1/*e*^2^).

Finally, when the probe only interacts with the negative-index time gradient, a pure blueshift (negative angular deflection) is clearly observable, which is longer in duration than the redshift phase. This is due to the asymmetric rise and fall times of the hot electron nonlinearities we are invoking. Figure 5b shows the theoretically calculated normalized angular intensity distribution. This second heat map is attained using the same numerical approach and parameters described in the ‘Spatio-spectral fission’ section, which were used for plotting the first row of the theoretical panels, where integration has been performed on both spectrum and the *y* coordinate. The set of five little icons in Fig. 5b represents the temporal delays corresponding to the spatio-spectral plots shown in Fig. 3. As we can see, the predicted angular deflection closely agrees with the underlying experimental data (note that Δ*t* = 3 fs is used in equation (9), which is consistent with the film thickness).

From the retrieved refractive-index profile, we can also estimate the maximum induced wavelength shifts to be 23 nm for the redshift and 15 nm for the blueshift, which is consistent with previously reported studies at slightly lower power levels^10^. However, when using our centre-of-mass metric for high incident angles, the spectral shifts become smaller than those directly predicted using our simplified model applied to a single peak wavelength.

## Conclusions

It is important to highlight that although significant efforts have been made to investigate the nonlinear modulation of spectrum and directionality of a propagating beam, this is the first time these two effects have been observed simultaneously on a macroscopic scale with comprehensive beam-reshaping distribution information. Besides the intrinsic significance of our combined spatio-spectral analysis, which opens new avenues for the temporal engineering of material properties, our work provides a deeper understanding of key findings recently reported in the literature. For instance, in ref. ^34^, although both spatial and spectral analyses are performed for a probe beam trespassing a time-varying layer (that is, an optically pumped ITO film), these were carried out by either integrating over space (that is, collecting all the transmitted light and sending it into a spectrometer) or by integrating over the spectrum (that is, by measuring optical power via colour-blind photodiodes)—approaches that might miss the overall complex spatio-spectral reconfigurability of the probe. To clarify this last point, we can also mention that if instead of performing a detailed spatially resolved spectral analysis of the probe, we had simply sent all the transmitted light into a spectrometer, the spatio-spectral fission reported in Fig. 3 (middle panels of the first and second rows) would have been misinterpreted as a simple spectral broadening. Another key factor arising from comparing our work with ref. ^34^ and other related works on the subject^8,15,16,30^ is that the fabrication conditions for maximizing the time-varying attributes of TCO films can be very critical. For instance, to attain spatio-spectral fission, a material that exhibits both ultrafast excitation and recombination times is required.

From equations (8) and (9), it is clear that our capability to temporally engineer the material for optical beam shaping and spectral redistribution is directly linked to the refractive-index time gradient. This parameter in the TCO is the highest possible with experimentally proven effective refractive-index change of about 0.5 in only a few femtoseconds (few-cycle pulse)^8^. In addition to this, due to the hybrid nature of the TCOs, the material response time can be forcefully shortened by the combined use of interband and intraband optical pumping^15^. Owing to the unprecedented nonlinear optical properties of low-index conducting oxides, we now have the freedom to transfer our capability in engineering the optical excitation (duration, shape and chirp of the pump) into the possibility to design a specific time-varying material.

In conclusion, we report on the splitting of a train of ultrafast 93-fs pulses centred at the telecom wavelength into two almost-equal parts as they trespass a submicrometre-thin time-varying layer of TCO. This sudden—and almost complete—split shows a significant angular separation of >1.5° (0.85° for a redshifted beam and −0.7° for a blueshifted beam), which is accompanied by a strong spectral fission and a large (>300%) transmission enhancement. A simplified model has also been developed that can quantitatively grasp both spectral and spatial time refractions, with the only fitting parameters being the temporal refractive-index profile induced by the pump and interaction time Δ*t*. From this analysis, a nonlinear index profile was recovered that closely matches those measured in previous reports and accurately fits our experimental data. Our results show how versatile low-index TCOs are for the temporal sculpturing of material optical properties. This is a new paradigm in material engineering in which attention is shifted away from standard fabrication processes (thin-film deposition and nanopatterning) and is focused instead on the design of a targeted temporal response by tailoring the most opportune ultrafast optical excitation (the pump wave). This study provides fundamental insights into the remarkable potential of these materials for applications in all-optical integrated photonics towards the exploration of photonic time crystals, on-chip generation of non-classical light and integrated neural networks.

## Methodology

### Experimental details

The pump-to-probe setup used 93-fs pulses at a repetition rate of 1 kHz. The optical parametric amplifier output was tuned to 1,300 nm, which corresponds to the epsilon-near-zero point of our 900-nm AZO film. The output is then reshaped using a telescope and is horizontally polarized. The beam is split in an 80:20 ratio, and the smaller part is used as the probe. The pump beam is focused onto the sample at normal incidence with a 10-cm lens, resulting in a 60-μm (1/*e*^2^) spot size with a peak power of 1.8 TW cm^–2^. The probe beam is attenuated to a power level that excludes self-action nonlinearities and then focused onto the sample at an angle of 65° with a 7.5-cm lens, producing a 30-μm (1/*e*^2^) spot size on the AZO film. Finally, the probe is measured using both a near-infrared camera and a fibre-coupled spectrometer at a focal length from the sample. The AZO film used in our experiment is 900 nm thick and was deposited onto the silica substrate in a low-oxygen environment by pulsed laser deposition. Further fabrication details can be found elsewhere^35^. The characterization of the sample’s refractive index can be found in another work^28^. Preliminary pump-to-probe tests performed on the bare substrate were used to clean up the reported results from any spurious nonlinear contribution that could come from the substrate. In this regard, it is worth mentioning that despite using a high pump intensity, any effect on the probe from the bare fused silica substrate was negligible. This should not be a surprise given that the nonlinear Kerr coefficient in AZO is over four orders of magnitude higher that that in silica. Moreover, the signal trespassing the 900 nm of AZO arrives considerably attenuated at the AZO–fused silica substrate interface, further limiting any nonlinear contribution from the substrate and making our analysis even more robust.

The value of 65° was chosen via experimental means by looking at the angle maximizing nonlinear beam deflection. The number is also consistent with similar values reported in the literature to maximize nonlinear effects in different TCOs^13,33^.

We should stress that the angle of incidence is very critical to achieve full spatio-spectral fission because it alters the relative time duration of the probe with respect to the material response (and pump duration). For this reason, there is an optimal angle that maximizes the temporal overlap between the material response *n*(*t*) and the probe temporal profile (Fig. 3, middle panel, third row). In this way, we ensure to maximize the fraction of the probe energy affected by the material nonlinearities (d*n*/d*t*). However, this condition must be balanced with another issue. In fact, if the incident angle is too large, we quench the transmission and the corresponding signal-to-noise ratio; however, if it is too narrow, then we reduce the nonlinear angular separation (equation (8) and appendix D in the Supplementary Information). Given the critical balance among all these factors, the optimal incidence is experimentally evaluated by testing which angle produces the strongest spatio-spectral separation, with an optimal signal-to-noise ratio.

Information about the refracted beams have been simultaneously collected using different tools such as a high-resolution infrared camera, fibre-coupled spectrometers and energy meters for multiple values of Δ*τ*. The spectrometer was mounted on a calibrated motorized stage that allowed for a spatial scan of the spectral distribution at given *x* values along the horizontal axis orthogonal to the probe propagation direction.

**Fig. 6:**
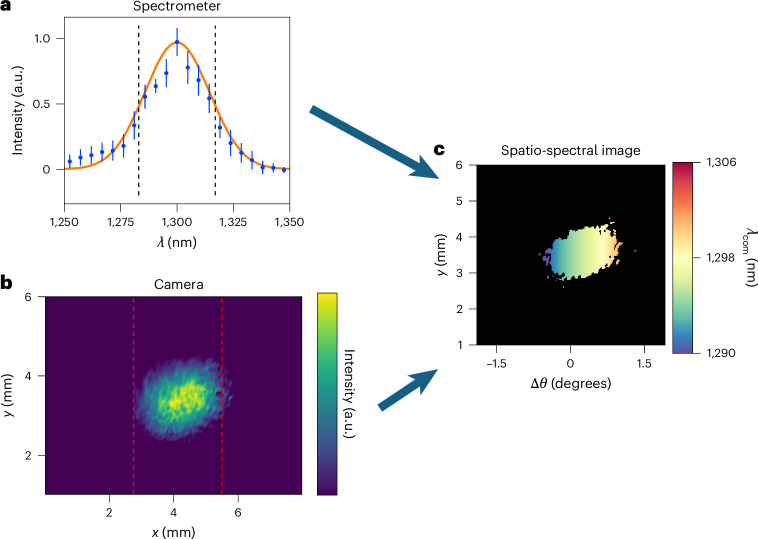
Process for creating spatio-spectral images from spectrometer measurements and camera images. **a**, Top left: example spectrum of the transmitted probe pulse for a given time delay Δ*τ*, and a given horizontal spatial position *x*. Data points with error bars represent mean values ± standard deviation as calculated from ten spectral measurements taken under identical experimental settings. **b**, Bottom left: spatial intensity profile as measured by a near-infrared camera. **c**, Right: full spatio-spectral profile of our probe pulse at a single pump-to-probe delay. The reported plots and images are taken for Δ*τ* = –206 fs. Dashed lines, in both **a** and **b**, define the spectral and spatial partitioning required for the analysis reported in the ‘Energy redistribution’ section.

From equations (8) and (9), we see that as the probe pulse trespasses the time-varying layer, both its spectrum and shape must be affected by the index time gradient. To provide a global picture of what happens to both energy and spectrum of the transmitted probe, the information acquired by both camera and spectrometer were combined. This unification was attained by assigning one unique wavelength to each *x* point, which was evaluated as the centre of mass of the corresponding spectrum. This analytical process is depicted for a given Δ*τ* in Fig. 6 where, in the lower panel, the spatial probe profile is mapped to its local spectral distribution. Here the *x* axis is converted into units of angular deflection using $\Delta \theta ={\tan }^{-1}\frac{x}{L}-{\theta }_{i}$, where *θ*_i_ is the angle of incidence and *L* is the distance from the sample to the camera, which we set at 7.5 cm.

### Numerical verification

A numerical analysis was completed to verify the infinitely thin-film approximation used in our simplified model in addition to the effect of including both losses and dispersion. This was done via a second-order finite-difference time-domain scheme for Maxwell’s equations coupled to the auxiliary differential equation method for modelling the AZO’s dispersion^36^. The nonlinear perturbation to the material was simulated by modifying the plasma frequency and damping constant in the Drude model at each time step in accordance with the index perturbation. The values for these parameterized changes were recovered from fitting multiple linear Drude models (for each simulation time step) to the pumped dispersions found in ref. ^28^. Finally, we interpolated this dispersion over a Gaussian index perturbation induced by a 93-fs pulse that propagates through our material. The finite-difference time-domain simulations and simplified model deliver very similar results. Additionally, to validate the existence of spatio-spectral fission when both losses and dispersion are considered, we have applied a more comprehensive material model. A detailed discussion about all the numerical verifications is provided in appendix F in the Supplementary Information.

## Online content

Any methods, additional references, Nature Portfolio reporting summaries, source data, extended data, supplementary information, acknowledgements, peer review information; details of author contributions and competing interests; and statements of data and code availability are available at 10.1038/s41566-025-01640-1.

## Supplementary information

Supplementary Information Supplementary Appendices A–F.

## Data Availability

All data generated in this study are available from the Heriot–Watt University database at 10.17861/be5becd5-19cf-48b1-b58b-c7257c65a2ac.
