# Supplementary material for: Spatio-spectral optical fission in time-varying subwavelength layers
Source: Nat Photonics. 2025 Mar 7;19(6):558–66. doi: 10.1038/s41566-025-01640-1 (PMC12141041; doi:10.1038/s41566-025-01640-1)
Supplement: Supplementary file 1 — Supplementary Appendices A–F. [file 41566_2025_1640_MOESM1_ESM.pdf]

nature photonics

Supplementary information

https://doi.org/10.1038/s41566-025-01640-1

Spatio-spectral optical fission in time-varying subwavelength layers

In the format provided by the authors and unedited

Supplementary materials

Appendix A - Pulse propagation through time-varying layers

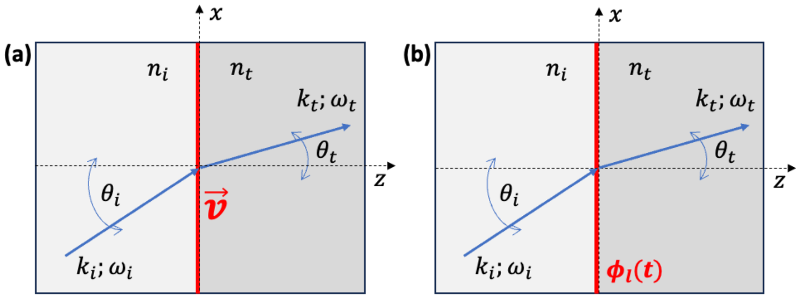

Fig. 1 - Spatio-temporal refraction and Snell's law adaptation. (a) Generalised case for an ideal merge between pure spatial and pure temporal refractions. (b) Intermediate model as described by Eq (5) in the main manuscript, which could adequately represent the case of a metallic metasurfaces on top of a nonlinear substrate.

Within the context of time varying materials, the dual case of an electromagnetic wave trespassing a spatial interface, is a radiation which experiences a sudden and global change of the index in time. While the former case pertains to the well-known Snell's law where energy is conserved (i.e., $\omega_1 = \omega_2$), the latter deals with the concept of time refraction, where instead momentum is the invariant parameter (i.e., $k_1 = k_2$).

A more general situation can be considered, where the medium is both non-uniform in space and non-stationary in time. When this condition is achieved, by combining ideal space and time refraction, an equivalent model can be considered where the interface is moving at a given speed $\vec{v}$ normal to the interface (see Fig.1-a) [1], [2]. The correspondent generalised Snell's law can be written as [3]:

$$n_1\omega_1 sin\theta_1 = n_2\omega_2 sin\theta_2 \qquad [1]$$

Where, n_1 and ω_1 are the index and frequency before the moving boundary and n_2 and ω_2 are the index and frequency after the moving boundary.

However, when the non-stationary system under analysis is a non-moving, and optically pumped, thin film of highly nonlinear low-index conductive oxide, the generalised and idealistic model proposed in [4] requires some adaptations. Indeed, the new model we seek should account for the deeply subwavelength nature of the system in use. In fact, at the lowest value of the refractive index, the operational effective wavelength in conductive oxides is several times longer than the film thickness, which is set to be sub-micron to mitigate the material losses. Now, considering the scenario represented in Fig.1b, a non-moving spatial interface, which is naturally defined by two materials with different static indices n_i and n_t, induces an additional time-varying phase term $\phi_l(t)$ to the trespassing radiation (represented by Eq. 5 in the main manuscript). Such a system is somewhat difficult to find in the real world, however, a metallic metasurface patterned on top of a dielectric nonlinear substrate could fulfil the criteria. For this device to operate as represented in Fig.1b, the nano-antennas should only act as field enhancers to localise the nonlinearities at the interface. An attempt to reproduce such system is reported in [5] where non-reciprocal behaviour is recorded for a beam reflected by an optically pumped silicon grating on a silica/silver bi-layer. However, the device used is

limited in bandwidth (thus tested in a quasi-monochromatic regime), requires complex material fabrication processes, and needs the combined effect of two beating pump waves to induce a relevant temporal phase change. A theoretical discussion of the equivalent system is reported in [3].

Appendix B – Nonlinearities in time-varying systems

Although standard optical nonlinearities can lead to spectral energy redistribution, they conceptually differ from the nonlinear events occurring in time-varying media, as the latter are non-resonant, not subjected to selection rules (i.e. phase matching), and exhibit a scattering cross section approaching unity (i.e. it will affect all propagating photons) [1]. All these subtle differences can be intuitively understood by starting (as usual) from the harmonic oscillator.

Let us consider the classical nonlinearities associated with bound electrons, which emerge from the anharmonic oscillations far from the equilibrium point. Consequently, nonlinear harmonics are generated only along a limited range of the electron motion. This oscillator can be modelled by the following Duffing equation [6]

$$\frac{d^2P}{dt^2} + \gamma\frac{dP}{dt} + \omega_0{}^2P + \beta(P \cdot P)P + \Theta(P \cdot P)^2P + \cdots = \omega_p{}^2E \qquad [2]$$

Here, E is the electric field, P is the polarisation of the material, γ is the damping frequency, ω_0 is the resonance frequency, ω_p is the plasma frequency, and β and Θ are first and second order tensors describing the nonlinear restoring force, respectively. This mathematical representation is similar to the standard polynomial expansion of material polarisation describing optical nonlinearities in bound electrons [7].

On the other hand, hot electron nonlinearities stem from changes of the electrons' effective mass [8], [9]. In fact, when pumping a metallic material, the electrons in the conduction band are heated up, thus modifying their effective mass, which in turn changes the plasma frequency, and ultimately modifies the refractive index. This is what happens in TCOs for a rather broad bandwidth around the cross-over wavelength (where the real permittivity changes sign). This model can be justified by considering a Drude dispersion model. Starting from the relation between polarisation **P**, susceptibility χ_D, and electric field **E**, we have:

$$\mathbf{P} = \chi_D\mathbf{E} \qquad [3]$$

And the Drude model's susceptibility χ_D is defined as:

$$\chi_D = -\frac{\omega_p^2}{\omega^2 + i\omega\gamma} \qquad [4]$$

Where ω is the frequency and γ is the damping constant. The plasma frequency ω_p is defined as $\omega_p = \sqrt{ne^2/\varepsilon_0 m_e}$, where n is the charge density, e is the electron charge, ε_0 is free space permittivity, and m_e is the electron effective mass. By combining the above equations, we arrive at:

$$-(\omega^2 + i\omega\gamma)\mathbf{P} = \omega_p^2\mathbf{E} \qquad [5]$$

Which after Fourier transforming provides us with the time domain polarisation response.

$$\frac{d^2\mathbf{P}}{dt^2} + \gamma\frac{d\mathbf{P}}{dt} = \omega_p^2\mathbf{E} \quad [6]$$

Finally, the strong hot electron nonlinearity present in TCOs can be approximated by considering a time varying electron effective mass $m_e(t)$ and therefore a time varying plasma frequency $\omega_p(t)$. This results in the equation below:

$$\frac{d^2\mathbf{P}}{dt^2} + \gamma\frac{d\mathbf{P}}{dt} = \omega_p^2(\mathrm{t})\mathbf{E} \quad [7]$$

This model is further approximated by considering a time varying index without any dispersion as we do in this work. This discussion justifies the general difference in handling free electron vs bound electron nonlinearities, however, the accurate modelling of the full optical properties of TCOs is a much more complicated process which is described subsequently in the present document in Appendix F.

In case of hot electron nonlinearities as described above, the nonlinearity is no longer directly dependent on the electric field and associated material polarisation, and the underlying process is always “active”, thus leading to a unitary scattering cross section. For all these reasons, Eq.(7) implicitly describes the nonlinear optical behaviour of TCOs and justifies why they are the best possible approximation to ideal time-varying systems [10], [11].

In these compounds, we depart from the usual local description of the material response related to electron-photon interaction, and instead consider light that is immersed in a uniform medium that changes its impedance with time. These differences between time refraction and more ”traditional” nonlinear optical processes, can lead to largely unexplored research territories extending into the domains of non-reciprocal systems [3], quantum optics [12] [13], photonic time crystals [11], [14], [15], [16], and beyond [17] [18], [19], [20]. A great part of the quantitative analysis that follows in the present manuscript, starts from an experimentally retrieved time profile of the refractive index, which is induced by an intense pump trespassing a thin film of AZO. The correspondent index time gradient will be directly linked to the observed spatial redistribution of spectral energy without any consideration on the microscopic material attributes leading to such extraordinary nonlinearities. For a deeper analysis of the nonlinear optical properties in TCOs we refer the reader to [21].

Appendix C – FROG measurement

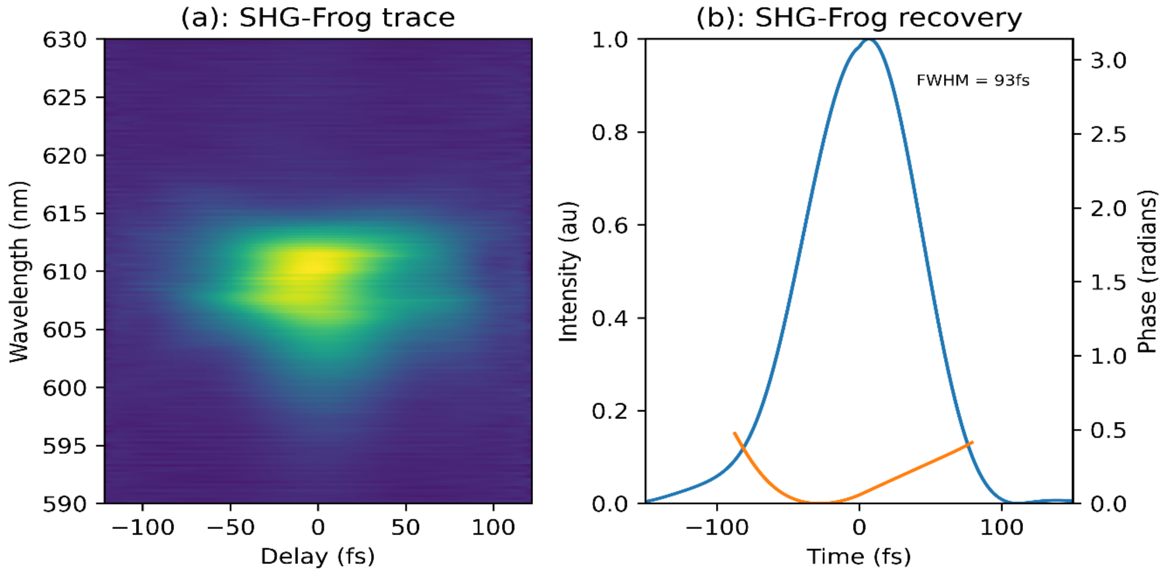

Fig. 2 – a) Measured SH FROG trace. b) Recovered pulse profile from SH trace using iterative FROG algorithm. Results indicate an unchirped gaussian pulse with duration of 93 fs.

In Fig. 2a above we report a SH FROG trace of the pulse used in this manuscript for both pumping and probing our AZO film. Using an iterative FROG algorithm, we recover our pulse's temporal profile in Fig. 2b and demonstrate that it is unchirped and has a gaussian shape with FWHM duration of 93 fs.

Appendix D – Angular shift vs incidence angle

Using Eq. (8) in the main manuscript, we map the angular shift $\Delta\theta$ against index change $(\partial n/\partial t)\Delta t$ and incident angle θ_i in Fig. 3. From this analysis, we can clearly see that operating at higher incident angles provides the strongest angular shifts. Additionally, the negative deflections are not as strong as the positive.

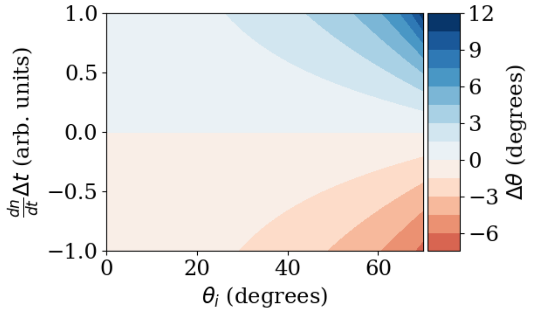

Fig. 3 – Angular shift due to time refraction vs index shift (the interaction time is included for clarity) and incident angle. At higher incident angles the angular shift becomes larger. An asymmetry in the positive and negative shift is also evident.

Appendix E – Spatial-spectral fission chirp consideration

Given that the probe nonlinear spectral redistribution is fundamentally linked to its temporal overlap with the material response (main manuscript Fig.3 central panel, third row), it is interesting to briefly discuss the effect of chirping the pulse (while keeping the same time duration) for the most extreme case of $\Delta\tau = 0$. As the linear index can be considered constant within the pulse bandwidth, the observed spatial split is expected to be almost unaltered by the chirp. However, important spectral changes are expected since chirping a pulse alters the temporal distribution of the harmonic content. For instance, for the case of a positive chirp (where the leading edge has more red frequencies and the back of the pulse has more blue frequencies) we expect to further enhance the frequency shift as the red portion of our pulse would be further red-shifted, and the blue would become more blue-shifted. For a negative chirp, where the front of our pulse includes more of the blue frequencies and the back more of the red frequencies, we expect a compensation effect, which should lead to a fission which is only spatial. In this case, both positively and negatively deflected pulses have equal spectra content. These considerations, although supported by applying our simplified model, still requires a deeper theoretical and experimental analysis for further validation.

Appendix F – Numerical considerations

Simplified model verification

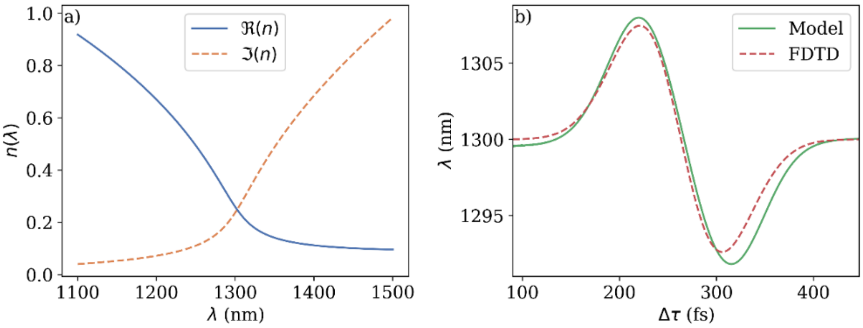

Fig. 4 – Numerical verification of wavelength shift in presence of dispersion and losses. a) Linear refractive index fit for the 900 nm AZO sample used in experiments. b) Wavelength shift predicted from the index perturbation using the simplified model vs a FDTD approach.

A second order FDTD scheme for Maxwells equations coupled to the (auxiliary differential equation) ADE method for modelling the AZO's dispersion [22]. The nonlinear perturbation of the film was simulated by modifying the plasma frequency and damping constant of a linear Drude model at each time step in accordance with the index perturbation. The values for these parameterised changes were recovered from fitting various linear Drude models at each instant of time to the pumped dispersions found in [23] (the unpumped dispersion fit for our sample is shown in Fig.4a). Finally, we interpolated this dispersion over a gaussian index perturbation of 93 fs that propagates through our material at the group velocity of the probe. The result of this comparison is shown in Fig. 4b, where both the FDTD simulation and simplified model deliver similar results. It is worth mentioning how remarkable is the agreement between theory and

experiments despite the simplicity of the model, which does not account for dispersion and losses, thus neglecting the effects that the nonlinear change in the imaginary index could have on both spatial and spectral redistribution. However, previously reported studies, looking into the complex nonlinearities of AZO films for very similar excitation conditions, show that although an increase of the real index is accompanied by a contextual reduction of the associated imaginary part (as also dictated by the Kramers-Kronig relations), the magnitude of the former is considerably larger than that of the latter, thus making our simplified analysis rather robust against experimental tests, as demonstrated by our FDTD test.

Material modelling

The theoretical description of macroscopic electrodynamics of conducting oxides begins with ellipsometric retrieval of the complex, local dielectric response of the sample. The complex function is then fitted using a Drude-Lorentz model having a resonance in the UV range, such that when combined with the Drude contribution yields:

$$\varepsilon_{AZO}(\omega) = 1 - \frac{\omega_{p,b}^2}{\omega^2 - \omega_{0,b}^2 + i\gamma_b\omega} - \frac{\omega_p^2}{\omega^2 + i\gamma_f\omega} \qquad [\mathbf{8}]$$

Here, $\omega_{p,b}$ is the bound electron plasma frequency, defined similarly to the free electron counterpart; $\omega_{0,b}$is the resonance frequency; and γ_b the bound electron damping coefficient. Fitting the retrieved, local dielectric constant helps us determine the damping coefficients, effective masses, and densities to be inserted in dynamical equations of motion that will replace Eq. (8) and allow us to study the dynamics. In what follows we report the most salient details of the hydrodynamic-Maxwell model that was used to describe second and third harmonic generation from an ITO nanolayer [9], and high harmonic generation from a 310nm-thick AZO layer [21], appropriately modified under the circumstances to model pump-probe dynamics. The material equations of motion that describe free and bound charges may be written as follows:

$$\ddot{\boldsymbol{P}}_f + \tilde{\gamma}_f\dot{\boldsymbol{P}}_f = \frac{n_{0,f}e^2\lambda_0^2}{m_f^*(T_e)c^2}\boldsymbol{E} + \frac{3E_F}{5m_f^*(T_e)c^2}\left(\nabla(\nabla \bullet \boldsymbol{P}_f) + \nabla^2\boldsymbol{P}_f\right) \qquad [\mathbf{9}]$$

$$\ddot{\boldsymbol{P}}_b + \tilde{\gamma}_b\dot{\boldsymbol{P}}_b + \tilde{\omega}_{0,b}^2\boldsymbol{P}_b + \boldsymbol{P}_{b,NL} = \frac{n_{0,b}e^2\lambda_0^2}{m_b^*c^2}\boldsymbol{E} \qquad [\mathbf{10}]$$

Eq. (9) and Eq. (10) describe the dynamics of free and bound electrons, respectively; T_e is the free electron temperature;

$$\boldsymbol{P}_{b,NL} = \alpha\boldsymbol{P}_b\boldsymbol{P}_b - \beta(\boldsymbol{P}_b \bullet \boldsymbol{P}_b)\boldsymbol{P}_b + \delta(\boldsymbol{P}_b \bullet \boldsymbol{P}_b)^2\boldsymbol{P}_b - \vartheta(\boldsymbol{P}_b \bullet \boldsymbol{P}_b)^3\boldsymbol{P}_b + \psi(\boldsymbol{P}_b \bullet \boldsymbol{P}_b)^4\boldsymbol{P}_b \qquad [11]$$

is the bulk crystal's nonlinear polarization; m_b^* is the bound electron mass; $n_{0,b}$ is the bound electron density, $E_F$is the Fermi energy, $\tilde{\gamma}_{f,b} = \gamma_{f,b}\lambda_0/c$, $\tilde{\omega}_{0,b}^2 = \omega_{0,b}^2\frac{\lambda_0^2}{c^2}$. Time and space have been scaled such that temporal and spatial derivatives are carried out with respect to the

following coordinates:$\varsigma = x/\lambda_0$,$\xi = z/\lambda_0$, and $\tau = ct/\lambda_0$. $\lambda_0 = 1$μmis a suitable reference wavelength.

The coefficients are also scaled accordingly, are to be interpreted as tensors, and carry information about crystal symmetry. For example, $\alpha = 0$ for centrosymmetric media like AZO, ITO and noble metals. Third and higher order nonlinear coefficients reflect the symmetry of an isotropic medium but can easily be generalized to describe other crystal symmetries. The combination of Eq. (9) and Eq. (10) preserve both linear and nonlinear dispersions. The polarization is expanded up to 9th order because incident peak power densities exceed 1TW/cm^2. The free electron component in AZO is characterized by a free electron mass m_f^* that is now a function of temperature. The second term on the right-hand side of Eq. (9) are nonlocal terms (pressure and viscosity.)

Hot carriers are usually described by implementing the two-temperature model, which couples the lattice temperature to that of the electron gas and is used to determine the instantaneous plasma frequency. However, if the electron temperature is only a few thousand degrees Kelvin one may assume that for a typical conductive oxide like AZO we can write:

$$m_f^*(T_e) \approx m_0^* + aK_BT_e = m_0^* + aK_B\Lambda \int\int \boldsymbol{J} \bullet \boldsymbol{E} d\boldsymbol{r}^3\, dt \qquad [12]$$

where K_B is Boltzmann's constant; a and Λ are constants of proportionality; m_0^* is the electron's rest mass for no applied field; $\Lambda \int\int \boldsymbol{J} \bullet \boldsymbol{E} d\boldsymbol{r}^3\, dt$represents absorption; and$\boldsymbol{J} = \dot{\boldsymbol{P}}_f$is the current density, the solution of Eq. (9). For simplicity we assume that$\boldsymbol{J} = \sigma_0\boldsymbol{E}$, where σ_0 is a constant to be determined. Using the temperature dependent expression for the effective mass Eq. (8), the leading term on the right-hand side of Eq. (9) may be written as:

$$\frac{n_{0,f}e^2\lambda_0^2}{m_f^*(T_e)c^2}\boldsymbol{E} = \frac{n_{0,f}e^2\lambda_0^2}{m_0^*c^2}\left(1 + \frac{\Lambda}{m_0^*}\int\int \boldsymbol{J} \bullet \boldsymbol{E} d\boldsymbol{r}^3 dt\right)^{-1} \boldsymbol{E} \approx$$
$$\frac{n_{0,f}e^2\lambda_0^2}{m_0^*c^2}\left(1 - \frac{\Lambda\sigma_0}{m_0^*}\int\int \boldsymbol{E} \bullet \boldsymbol{E} d\boldsymbol{r}^3 dt + \left(\frac{\Lambda\sigma_0}{m_0^*}\right)^2 \left(\int\int \boldsymbol{E} \bullet \boldsymbol{E} d\boldsymbol{r}^3 dt\right)^2 + \cdots\right)\boldsymbol{E} \qquad [13]$$

The effective mass appears in both terms on the right-hand side of Eq. (9). However, only the linear terms survive the expansion. For simplicity we may also drop the volume integral in Eq. (13) and introduce a parameter $\tilde{\Lambda}$ proportional to the product of$\frac{\Lambda\sigma_0}{m_0^*}$, the interaction volume and the temporal duration of the pulse, so that Eq. (14) takes the following, simplified form:

$$\frac{n_{0,f}e^2\lambda_0^2}{m_f^*(T_e)c^2}\boldsymbol{E} \approx \frac{n_{0,f}e^2\lambda_0^2}{m_0^*c^2}\boldsymbol{E} - \tilde{\Lambda}(\boldsymbol{E} \bullet \boldsymbol{E})\boldsymbol{E} + \tilde{\Lambda}^2(\boldsymbol{E} \bullet \boldsymbol{E})^2\boldsymbol{E} - \tilde{\Lambda}^3(\boldsymbol{E} \bullet \boldsymbol{E})^3\boldsymbol{E} + \tilde{\Lambda}^4(\boldsymbol{E} \bullet \boldsymbol{E})^4\boldsymbol{E} \qquad [14]$$

The scaled coefficient $\tilde{\Lambda}$ thus combines information about fluence, conductivity, dispersion and determines the spatio-temporal dynamics of the redshift impressed upon the plasma frequency. Without loss of generality, the description of the interaction of two fields having arbitrary carrier wavelengths may be accomplished by assuming a TM-polarized incident field. The **E**

field has components in the y- (transverse) and z- (longitudinal) directions, while the **H** field is polarized along the x-direction, and may be written as follows:

$$\boldsymbol{E} = \boldsymbol{j}E_{\hat{y}} + \boldsymbol{k}E_{\hat{z}} = \boldsymbol{j}\left(E^{\omega 1}_{TM\hat{y}}(r,t)e^{i(k1\bullet r-\omega 1t)} + +E^{\omega 2}_{TM\hat{y}}(r,t)e^{i(k2\bullet r-\omega 2t)}c.c.\right)$$
$$+\boldsymbol{k}\left(E^{\omega 1}_{TM\hat{z}}(r,t)e^{i(k1\bullet r-\omega 1t)} + E^{\omega 2}_{TM\hat{z}}(r,t)e^{i(k2\bullet r-\omega 2t)} + c.c.\right) \qquad \textbf{[15]}$$

$$\boldsymbol{H} = \boldsymbol{i}H_{\hat{x}} = i\left(H^{\omega 1}_{TM\hat{x}}(r,t)e^{i(k1\bullet r-\omega 1t)} + c.c. + H^{\omega 2}_{TM\hat{x}}(r,t)e^{i(k2\bullet r-\omega 2t)} + c.c.\right) \qquad \textbf{[16]}$$

The polarization field $\boldsymbol{P}_b$is expanded similarly to the electric field. The preservation of all spatial and temporal derivatives accounts for dynamical changes to the instantaneous phases and amplitudes of the fields. Using the solutions of Eq. (9) and Eq. (10) the total polarization is written as the vector sum of free and bound electron contributions,$\boldsymbol{P} = \boldsymbol{P}_f + \boldsymbol{P}_b$, and is inserted into Maxwell's equations:

$$\boldsymbol{\nabla} \times \boldsymbol{E} = -\frac{1}{c}\frac{\partial \boldsymbol{H}}{\partial t}; \quad \boldsymbol{\nabla} \times \boldsymbol{H} = \frac{1}{c}\frac{\partial \boldsymbol{E}}{\partial t} + \frac{4\pi}{c}\frac{\partial \boldsymbol{P}}{\partial t} \qquad [17]$$

Wave propagation analysis

The integration of coupled equations Eq. (9), Eq. (10) and Eq. (17) in time is carried out using the well-known split-step, beam propagation algorithm modified to account for reflections [24]. The spatial derivatives of the fields are calculated using fast Fourier transforms (FFTs) by means of hundreds of thousands of plane waves in a two-dimensional spatial grid, while the polarization equation is integrated using a second-order accurate predictor-corrector method. The method of solution is described in exhaustive details in reference [6]. Expansion of the above expressions up the 9th power generates thousands of terms. However, we assume that the pump intensity is several orders of magnitude more intense than the probe field intensity, which reduces the number of terms to a few dozens, diminishing the complexity of the equations and facilitating the numerical integration process.

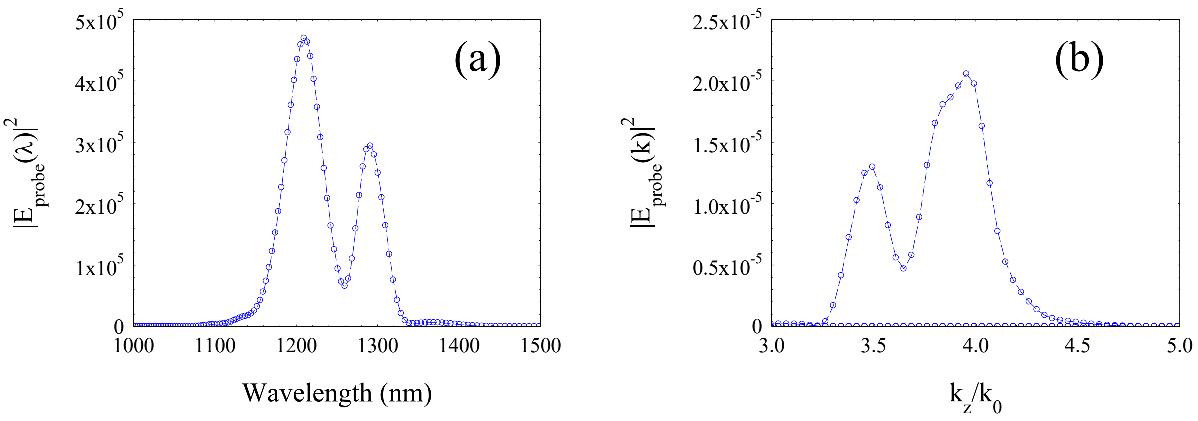

Fig. 5: (a) Power spectrum of the transmitted probe pulse collected a few microns after the second interface. The temporal profile of the transmitted field is collected and Fourier transformed to reveal the double-peaked structure corresponding to the two colors we observe in our experiment. The relative amplitudes of the two peaks are determined either by varying the peak power density, or the magnitude of the coefficients. (b) The transmitted spatial profile of the probe pulse is Fourier transformed, and the spectral density plotted as a function of the longitudinal component of the wave vector normalized by the incident wave vector. Both pump and probe pulses are approximately 85fs in duration, peak power density is ~1.75TW/cm^2, $\alpha = 0$,$\beta = 10^{-11}$,$\delta = 10^{-22}$,$\vartheta = 10^{-33}$,$\psi = 10^{-44}$, $\tilde{\Lambda} = 4.2 \times 10^{-11}$.

In Fig. 5a we plot the power spectra of the transmitted probe field, both characterized by corresponding double-peaked structure. Based on our initial conditions, it is possible to retrieve phase front refraction. An examination of Fig. 5b reveals the directions of k_1 and k_2, which are shown in Fig. 6 below. The dynamics are robust with respect to the relative delay of the peaks of the pulses, which is to say that increasing or decreasing the pump-probe delay by 10-15 fs does not disrupt the observed splitting dynamics.

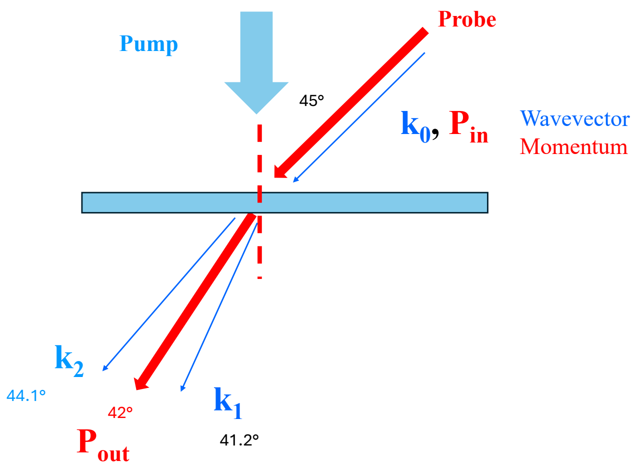

Fig. 6: The pump beam is incident normal to the 900-thick AZO sample and is seven orders of magnitude more intense than the probe beam, which in this case is incident at 45°. The pump strongly modulates the dielectric function (index of refraction) that both it and the probe experience, ultimately resulting in the alteration of the direction of propagation of the probe beam, as shown in the figure.

References

[1] J. T. Mendonca, *Theory of Photon Acceleration*. 2001. doi: 10.1887/0750307110.

[2] M. Koivurova, C. W. Robson, and M. Ornigotti, "Time-varying media, relativity, and the arrow of time," *Optica*, vol. 10, no. 10, pp. 1398–1406, 2023, doi: 10.1364/OPTICA.494630.

[3] A. Shaltout, A. Kildishev, and V. Shalaev, "Time-varying metasurfaces and Lorentz non-reciprocity," *Opt Mater Express*, vol. 5, no. 11, 2015, doi: 10.1364/ome.5.002459.

[4] E. Galiffi *et al.*, "Photonics of time-varying media," 2022. doi: 10.1117/1.AP.4.1.014002.

[5] X. Guo, Y. Ding, Y. Duan, and X. Ni, "Nonreciprocal metasurface with space–time phase modulation," *Light Sci Appl*, vol. 8, no. 1, 2019, doi: 10.1038/s41377-019-0225-z.

[6] M. Scalora, M. A. Vincenti, D. de Ceglia, C. M. Cojocaru, M. Grande, and J. W. Haus, "Nonlinear Duffing oscillator model for third harmonic generation," *Journal of the Optical Society of America B*, vol. 32, no. 10, p. 2129, Oct. 2015, doi: 10.1364/JOSAB.32.002129.

[7] R. W. Boyd, *Nonlinear Optics, Third Edition*. Elsevier, 2008. Accessed: May 15, 2024. [Online]. Available: http://www.sciencedirect.com:5070/book/9780123694706/nonlinear-optics

[8] J. B. Khurgin, M. Clerici, and N. Kinsey, "Fast and Slow Nonlinearities in Epsilon-Near-Zero Materials," *Laser Photon Rev*, vol. 15, no. 2, p. 2000291, Feb. 2021, doi: 10.1002/LPOR.202000291.

[9] L. Rodríguez-Suné *et al.*, "Study of second and third harmonic generation from an indium tin oxide nanolayer: Influence of nonlocal effects and hot electrons," *APL Photonics*, vol. 5, no. 1, 2020, doi: 10.1063/1.5129627.

[10] W. Jaffray *et al.*, "Near-zero-index ultra-fast pulse characterization," *Nat Commun*, vol. 13, no. 1, 2022, doi: 10.1038/s41467-022-31151-4.

[11] E. Lustig *et al.*, "Time-refraction optics with single cycle modulation," *Nanophotonics*, vol. 12, no. 12, 2023, doi: 10.1515/nanoph-2023-0126.

[12] J. T. Mendonça, A. Guerreiro, and A. M. Martins, "Quantum theory of time refraction," *Phys Rev A*, vol. 62, no. 3, 2000, doi: 10.1103/PhysRevA.62.033805.

[13] J. T. Mendonça and A. Guerreiro, "Time refraction and the quantum properties of vacuum," *Phys Rev A*, vol. 72, no. 6, p. 063805, Dec. 2005, doi: 10.1103/PHYSREVA.72.063805/FIGURES/4/MEDIUM.

[14] Y. Sharabi, E. Lustig, and M. Segev, "Topological aspects of photonic time crystals," *Optica, Vol. 5, Issue 11, pp. 1390-1395*, vol. 5, no. 11, pp. 1390–1395, Nov. 2018, doi: 10.1364/OPTICA.5.001390.

[15] M. Lyubarov, Y. Lumer, A. Dikopoltsev, E. Lustig, Y. Sharabi, and M. Segev, "Amplified emission and lasing in photonic time crystals," *Science (1979)*, vol. 377, no. 6604, pp. 425–428, Jul. 2022, doi: 10.1126/SCIENCE.ABO3324/SUPPL_FILE/SCIENCE.ABO3324_SM.PDF.

[16] A. Dikopoltsev *et al.*, "Light emission by free electrons in photonic time-crystals," *Proc Natl Acad Sci U S A*, vol. 119, no. 6, p. e2119705119, Feb. 2022, doi: 10.1073/PNAS.2119705119/SUPPL_FILE/PNAS.2119705119.SAPP.PDF.

[17] J. T. Mendonça, A. M. Martins, and A. Guerreiro, "Temporal beam splitter and temporal interference," *Phys Rev A*, vol. 68, no. 4, 2003, doi: 10.1103/PhysRevA.68.043801.

[18] H. Moussa, G. Xu, S. Yin, E. Galiffi, Y. Ra'di, and A. Alù, "Observation of temporal reflection and broadband frequency translation at photonic time interfaces," *Nature Physics 2023 19:6*, vol. 19, no. 6, pp. 863–868, Mar. 2023, doi: 10.1038/s41567-023-01975-y.

[19] S. Vezzoli *et al.*, "Optical Time Reversal from Time-Dependent Epsilon-Near-Zero Media," *Phys Rev Lett*, vol. 120, no. 4, 2018, doi: 10.1103/PhysRevLett.120.043902.

[20] V. Bruno *et al.*, "Negative Refraction in Time-Varying Strongly Coupled Plasmonic-Antenna-Epsilon-Near-Zero Systems," *Phys Rev Lett*, vol. 124, no. 4, 2020, doi: 10.1103/PhysRevLett.124.043902.

[21] W. Jaffray *et al.*, "High-Order Nonlinear Frequency Conversion in Transparent Conducting Oxide Thin Films," *Adv Opt Mater*, vol. 12, no. 28, p. 2401249, 2024, doi: https://doi.org/10.1002/adom.202401249.

[22] J. W. Banks *et al.*, "A high-order accurate scheme for Maxwell's equations with a Generalized Dispersive Material (GDM) model and material interfaces," *J Comput Phys*, vol. 412, 2020, doi: 10.1016/j.jcp.2020.109424.

[23] W. Jaffray, M. Clerici, B. Heijnen, A. Boltasseva, V. M. Shalaev, and M. Ferrera, "Nonlinear Loss Engineering in Near-Zero-Index Bulk Materials," *Adv Opt Mater*, vol. 12, no. 1, 2024, doi: 10.1002/adom.202301232.

[24] M. Scalora and M. E. Crenshaw, "A beam propagation method that handles reflections," *Opt Commun*, vol. 108, no. 4–6, 1994, doi: 10.1016/0030-4018(94)90647-5.
